# Elicitation of preferences in the second half of the shared decision making process needs attention; a qualitative study

**DOI:** 10.1186/s12913-020-05476-z

**Published:** 2020-07-09

**Authors:** W. Savelberg, M. Smidt, L. J. Boersma, T. van der Weijden

**Affiliations:** 1 Department of Quality and Safety, Maastricht University Medical Centre, P. Debyelaan 25, 6229 HX Maastricht, The Netherlands; 2 Care and Public Health Research Institute (CAPHRI), Maastricht University, Universiteitssingel 40, 6229 ER Maastricht, The Netherlands; 3 Oncology Centre, Maastricht University Medical Centre, P. Debyelaan 25, 6229 HX Maastricht, The Netherlands; 4 GROW School for Oncology and Developmental Biology, Department of Radiotherapy (MAASTRO clinic), Maastricht University Medical Centre, Dr. Tanslaan 12, 6229 ET Maastricht, The Netherlands; 5 Department of Family Medicine, Maastricht University, Debyeplein 1, 6229 ER Maastricht, The Netherlands

**Keywords:** Shared decision making, Patient decision aid, Early stage breast cancer, Implementation, Patient experiences

## Abstract

**Background:**

It is known that the use of a Patient Decision Aid (PtDA), combined with advice for professionals on how and when to use it, can enhance the involvement of patients in the treatment decision. However, we need more knowledge with respect to the intention-behaviour gap. This study aims to analyse patients’ experiences with the Shared Decision Making (SDM) process to find clues to close this gap.

**Methods:**

This qualitative study was part of a pilot study aiming to implement SDM in early adopter breast cancer teams. Patients were given access to a personalised PtDA. Breast cancer teams were instructed on how and when to deliver the PtDA. We interviewed 20 patients about their experience with the PtDA and SDM in general.

**Results:**

Most patients experienced SDM, though to a certain extent. Choice talk and option talk were commonly experienced, however the elicitation of preferences and decision talk was rare. The PtDA was used by the majority of patients (*N* = 13), all indicating that it was useful, especially to recall all the information given. Patients appreciated the contribution of breast cancer nurses in the SDM process. They considered them as true case managers, easy to approach and supportive.

**Conclusion:**

Although patients felt well-informed and satisfied about risk-communication, the elicitation of preferences appeared very limited to non-existent. We recommend that breast cancer teams divide tasks in the SDM process and reallocate the elicitation of preferences to the nurses in a well-defined clinical pathway.

## Background

A large number of women with early stage breast cancer are faced with the choice between breast conserving or breast removing treatment. This is a preference sensitive decision, as both options have similar survival rates [1], while each patient may value the advantages and disadvantages of both therapies differently. Therefore, the patient’s preferences and values play an important role in the decision making process [2].

In practice, however, treatment choices appear to be determined largely by local medical opinion [3]. Variation in hospital mastectomy rates is associated with differences in the consultation and decision-making experiences of breast cancer patients [4]. Patients’ decisions are affected by the treatment options they were offered, the content and style of the information provided by their clinicians, the level of patient autonomy, and the time provided for decision making [4].

Shared decision making (SDM) is increasingly seen as a promising model to involve patients in deciding about treatment when there is more than one option available. SDM is a process in which health care professionals and patients discuss treatment options, based on clinical evidence and the preferences of informed patients. First, the professional informs the patient that a decision is to be made and that the patient’s opinion is important (choice talk), then the professional provides evidence-based, neutrally framed information about options, treatment burden, benefits and side effects (option talk). Finally, clinicians support the patient to deliberate about the options, which involves exploring and considering preferences (preference talk) and deciding what fits the patient best (decision talk) [5, 6].

Despite clinicians reporting positive attitudes and the intention to apply SDM, it appears that the actual application of SDM during clinical encounters is limited [7]. Barriers for the implementation of SDM are found on the side of both clinicians as well as patients. Although the majority of patients prefer the SDM model, a minority of patients may not willing to be involved in the decision making process [8, 9]. Moreover, some patients feel a lack of capacity or knowledge to be involved in the decision making process [10–12]. Emotional distress could also prevent patients from engaging in the SDM process as well as taking the time needed to get involved [13]. However, given the significant positive correlation between active involvement in the decision making and satisfaction with the final decision, patients’ involvement in the decision-making process should be stimulated [14–16]. If SDM is applied correctly and carefully, this can lead to a decision that fits the patient, which not only results in more knowledge about options, increased satisfaction and less decisional conflict, but also in a better doctor-patient relationship, fewer repeat visits and fewer second opinions [6, 17, 18].

To implement SDM, we developed multifaceted strategies to enable breast cancer care teams to apply SDM, which included a web-based PtDA [19, 20], a motivational five-minute video for patients, a ten-minute educational video for professionals and tailored advice on when and how to indicate and disseminate the PtDA. We also advised teams on how to organize the care pathway to facilitate the SDM process, e.g. how to structure the different SDM steps in at least two different consultations.

Although clinicians report positive attitudes and the intention to apply SDM, the actual application of SDM during clinical encounters is limited. This study aimed to find clarification on the intention-behaviour gap from patient points of view by analysing: 1) the experiences of patients who were exposed to implementation strategies with regard to the SDM process; 2) the support patients experienced with regard to the PtDA.

## Methods

This study was part of a larger study on the implementation of SDM using a PtDA for the surgical treatment of women with early-stage breast cancer (stage I or II) [21]. The study took place from April 2015 until June 2017.

### Design

We conducted qualitative interviews to assess experiences from patients exposed to the implementation strategies to enhance SDM in breast cancer care. We used survey data on patient’s experiences with regard to the SDM process and the support they experienced regarding the PtDA to underpin the interview guide.

### Setting and population

Data collection took place in dedicated breast cancer teams in four Dutch hospitals and one specialist breast cancer hospital in the west and south east of the Netherlands between 1 April 2015 and 31 December 2015. These teams expressed their interest our study within the national network of breast cancer. We asked each hospital to recruit 10 patients (mother sample), as a number of 30 to 40 patients is recommended for a pilot study [22], using purposive sampling [23]. Patients had to be newly diagnosed with early-stage breast cancer, eligible for breast-conserving therapy or mastectomy and have been offered the PtDA. Eligible patients should be able to speak and understand Dutch and were identified at the tumour board meetings. The patients were asked to fill in four quantitative questionnaires. Breast cancer nurses were instructed to invite each included patient from the mother sample for an interview, until a minimum of four patients per hospital were included. We aimed to include patients until saturation was reached, resulting in sufficient in-depth information to recognize patterns, categories and variety [24].

### Implementation strategies

All the members of the breast cancer teams were invited to attend a meeting, which took place within their own hospital. The meeting was prepared by the research team, with the first author (WS) having contacted key members of the team in advance. During the meeting, the research team clarified the principles of SDM and the PtDA, if necessary, and explained the implementation strategies. The strategies existed of recommendations (tailored to the specific needs of the team and to the hospital’s specific workflow) on: 1. a procedure on when and how to present the PtDA to the patient; 2. minor adjustments to the pathway, such as planning a follow-up contact after patients used the PtDA; 3. watching both a ten-minute educational lecture and a five-minute motivational video on SDM (role modelling) and 4. a personalised web-based PtDA for patients. Participating patients were given access to the PtDA by means of a ‘prescription pad’. This prescription pad contained the website address and a unique login code. On each prescription pad, the professional ticks the pre-printed treatment options that are indicated for the individual patient. A link enables the patient to personalize the PtDA, by clicking the different buttons for treatment options as they are ticked on the prescription pad. The breast cancer nurses were to provide additional information on why and how to use the PtDA [19, 25]. Breast cancer nurses also informed patients about the present study and provided written information about the aim and procedure of the study, an informed consent form, three questionnaires and a prepaid envelope.

### Ethics

The Maastricht University Medical Centre (MUMC+) ethics committee declares that this study does not fall under the scope of the Medical Research Involving Human Subjects Act (METC 14–5-042). Handling of personal data was in accordance with the Dutch Personal Data Protection Act and Medical Research (Human Subjects) Act. All participants provided written informed consent and all data were processed confidentially and anonymously.

### Data collection

We collected demographic data such as age and level of education which we defined as low (early childhood education, primary education and lower secondary education), intermediate (upper secondary education, postsecondary non-tertiary education and short-cycle tertiary education) and high (bachelor’s or equivalent level, master’s or equivalent level, doctor or equivalent level) [26] and asked all patients we interviewed about their current or past occupation. We also measured knowledge, decisional conflict and the SDM process as perceived by patients. We used an adapted breast cancer information test to measure knowledge 1 week after the encounter [27]. Patients’ experience of the process of SDM was also measured at that time. We used the nine-item Shared Decision-Making Questionnaire (SDM-Q9) with a six-point scale from 0 (*completely disagree*) to 5 (*completely agree*), which is a standardised brief patient report instrument [28] used in clinical encounters, and the CollaboRATE which contains three brief questions with a 10-point anchored scale (0 = no effort was made, 9 = every effort was made) [29]. Patients eligible for this study received all three questionnaires, including a return envelope from the breast cancer nurse at the end of the last consultation before treatment started. To further characterise the patients, we also measured decisional conflict 3 months after their diagnosis. The patients received a questionnaire by post containing the Decisional Conflict Scale (DCS), a16-item scale capturing to which extent patients feel uncertain, feel uninformed, feel supported and are unclear about personal values. Each of these items is scored on a five-point Likert scale from 1 (*strongly agree*) to 5 (*strongly disagree*) and summed to a total score of 1–100, with higher scores corresponding to more decisional conflict [30]. These patient-reported experiences on the process of SDM were used to enrich the interview guide (Appendix 1). The average score for patients in the mother sample on their experience of SDM was 86.3 (SD 12.2 range 0–100, whereby 0 indicates the lowest possible level of SDM and 100 indicates the highest possible level of SDM). Their mean knowledge score was 61% (SD 15 proportion of correct answers) (Table 1).

**Table 1:** Results of the quantitative survey data in five hospitals

	Total	
	Patients interviewed (*N* = 20)	Mother sample (*N* = 41)
✓ SDM-Q9 Mean (SD)	87.4 (11.2)	86.3 (12.2)
✓ CollaboRATE Mean (SD)	8.2 (0.5)	8.3 (0.6)
✓ Knowledge Mean (SD)	65% (14.1)	61% (15)
✓ Decisional conflict Mean (SD)	31.6 (10.8)	28.1 (11.4)

SDM-Q9: range 0–100, whereby 0 indicates the lowest possible level of SDM and 100 indicates the highest possible level of SDM [28].

CollaboRATE: Top score = percentage of the patients responding 9 to all three items. Higher scores represent more shared decision-making [29].

Knowledge ranges from 0 to 100% depending on the number of correct answers.

The survey data were entered into a SPSS database, checked for completeness and errors by randomly controlling 15% of the data, and analysed using descriptive statistics (frequencies and percentages). We analysed these data and used them to enrich our interview guide.

All included patients in the mother sample were invited to participate in a semi-structured face-to-face interview. If a patient gave permission to be approached, the researcher (WS) called them within 2 months after diagnosis to make an appointment for the interview, to be conducted at their home or in the hospital. The interviews systematically addressed the following subjects: 1) experiences and satisfaction with the process of SDM; 2) the usefulness and the degree of support provided by the PtDA. The interviews were conducted by a trained fifth-year medical student or the researcher (WS) and were audio-recorded. An interview guide was used. The interviewer took field notes.

### Data analysis

For the qualitative data, the interviews were transcribed verbatim and independently coded by the first author and a trained fifth-year medical student, using NVivo software to organise the data. The qualitative content analysis was performed using direct content analysis [31]. We identified key themes that appeared throughout all interviews by grouping the codes into larger themes: 1) perceived SDM and SDM steps (choice talk, option talk and decision talk, including elicitation of preferences) during the clinical encounters; 2) delivery of the PtDA (by whom, how and when the PtDA was presented to the patient); 3) perceived usefulness of the PtDA. Differences in opinion were solved by discussion until agreement was reached.

## Results

### Study population

**Fig. 1:**
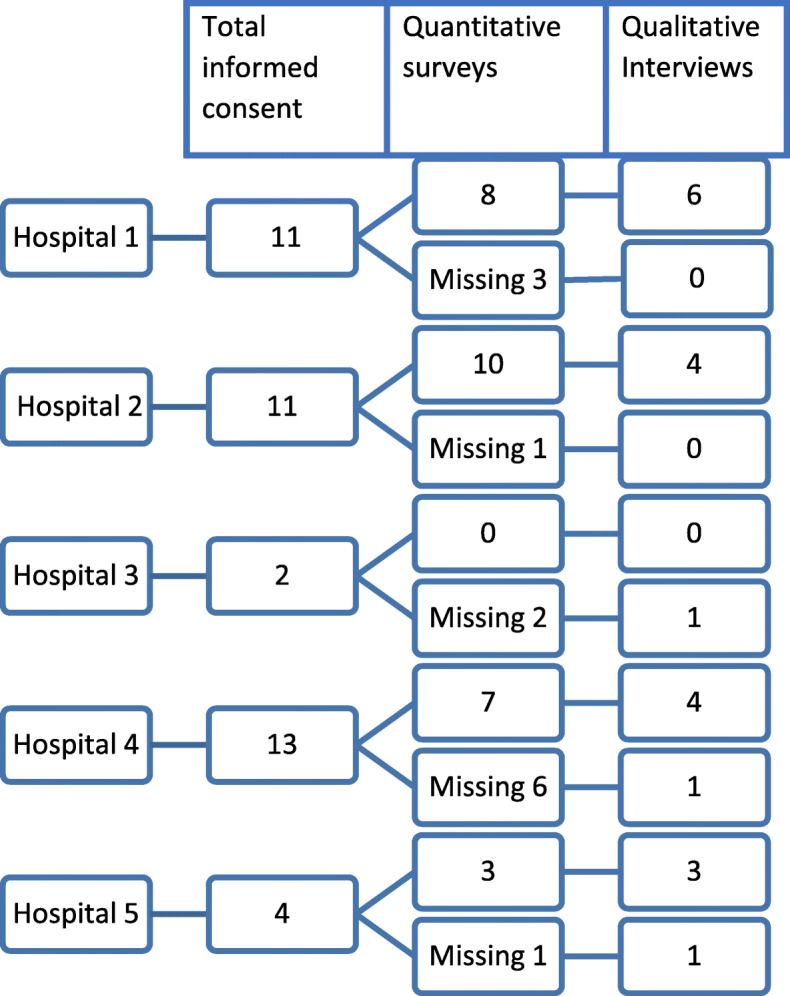
overview of the sample selection

We gathered data from 41 patients, (Fig. 1) of whom 20 agreed to be interviewed, from two hospitals we interviewed 4 patients, from the other 3 hospital we interviewed 1, 5 and 6 patients respectively. We interviewed these patients within 3 months of the diagnosis; the median time from diagnosis until the interview was 10 weeks (range 8–13 weeks). From these 20 patients, survey data were available for 17 patients.

For different reasons, e.g. no eligible patients, no time or protection of patients overburdened by research, patient inclusion for the mother sample was difficult resulting in scattered and missing data. Nevertheless, the data made us aware of overarching potential issues, such as a perceived feeling of being well-informed and problems with some of the steps of SDM. This resulted in the interview guide directing to dig deeper into the experience of patients with regard to preference and decision talk.

The 20 interviewed patients were between 44 and 68 years of age with an average of 53 (SD 9.7) years, which did not differ from the patients in the mother sample with an average of 54 (SD 10.1). The interviews lasted between 11 and 67 min, with an average of 37 min. Of the 20 interviews, 14 were conducted face to face and six by phone. Table 2 describes the patient characteristics.

**Table 2:** Patient characteristics

Patients	Hospital	Surgical treatment at time of interview	Age	Level of education	Logged in PtDA	Interview length in minutes	DCS^a^ ^b^
Patient 1	1	Mastectomy	44	High	Yes	32	10.9
Patient 2	2	Lumpectomy	58	Inter-mediate	No	31	28.1
Patient 3	2	Lumpectomy	50	Low	Yes	54	^c^
Patient 4	1	Lumpectomy	55	High	Yes	41	31.3
Patient 5	1	Depending on chemo response	48	Inter-mediate	No	34	^c^
Patient 6	1	Mastectomy	56	Inter-mediate	No	47	29.7
Patient 7	1	Lumpectomy	59	High	Yes	46	^c^
Patient 8	3	Lumpectomy Mastectomy	44	Inter-mediate	No	46	^c^
Patient 9	2	Lumpectomy	44	Inter-mediate	Yes	11	26.6
Patient 10	2	Lumpectomy	61	High	Yes	29	12.5
Patient 11	1	Lumpectomy	51	Low	No	32	26.6
Patient 12	5	Mastectomy	46	Inter-mediate	Yes	13	^c^
Patient 13	5	Mastectomy	61	High	Yes	53	^c^
Patient 14	4	Lumpectomy	66	Low	No	11	15.6
Patient 15	5	Lumpectomy	51	Low	No	16	32.8
Patient 16	5	Lumpectomy	68	High	Yes	35	^c^
Patient 17	4	Lumpectomy	51	High	Yes	45	9.8
Patient 18	4	Lumpectomy	51	High	Yes	27	45.3
Patient 19	4	Lumpectomy	50	Inter-mediate	Yes	57:	26.6
Patient 20	4	Lumpectomy	53	High	Yes	28	26.6

^a^*DCS* Decisional conflict scale ^b^Total score 1–100 ^c^Missing

With regard to the interviews, It appeared that patients with a higher level of education reflected more extensively on the conversation with their clinicians. They remembered with greater detail what their clinician told them and what questions they posed, like what the impact of the treatment would be on their job. This seemed to be in contrast to patients with low level of education, who reported discussing more general or practical issues, such as the different steps until the treatment, without getting into too much detail.

#### Experienced SDM steps and satisfaction with the SDM process.

In general, patients reported to have experienced SDM and were positive with regard to this experience of patient-centeredness. Most patients were positive about the process, the information they received, the way the clinicians supported and guided them and their approachability. Some patients experienced patient-centeredness when health care professionals showed a true personal interest in them.

Most patients said they received a lot of information. Some patients found this somewhat overwhelming and stated that they forgot most of the information. Nevertheless, they generally felt well-informed and well taken care of. The effort of the breast cancer nurses was especially appreciated. *‘They record the things that you are concerned with, and then they bring up these topics themselves.’ … ‘Those are the sorts of little things that make it so pleasant.’ Patient 1*

*‘You receive tremendous amounts of information, and I truly believe they really tell you everything, but obviously you don’t absorb it all.’ Patient 6*

*‘I could always give her a call and turn to her with any questions I had or if I was feeling really lost.’ Patient 3*

### Choice talk

Although some patients experienced a more paternalistic decision process, most patients reported that the clinicians, mostly the surgeon, made them aware about two relevant treatment options. Most of the women remembered that their clinician specifically mentioned that both treatment options were equal with regard to survival rates and recurrence.

*‘Within five minutes, I was told that it was breast cancer and that there were two options: breast-conserving therapy or breast amputation.’ Patient 17*

### Option talk

Most of the patients reported the surgeon giving a comprehensive explanation of the tumour (size, location, risk of metastasis) and also describing the treatment options in general terms. Some patients referred to the surgeon also pointing out the pros and cons of both options. Occasionally a patient actively remembered how one of the clinicians summarised the options. Some patients received additional information from the breast cancer nurse in a second consultation.

*‘And then he just began telling me what the pros and cons were of the various treatment options.’ Patient 5*

*‘Although they said that the survival chances were equal with breast-conserving or … for me total amputation really felt strongly like, well … with breast conserving-therapy, you never know what you leave behind.’ Patient 12*

### Preference talk

A few patients indicated that they were asked about their preference for one of the options, after the clinician informed them about these options. In most cases, patients did not experience a discussion on preferences or any prompting for value clarification. A few patients expressed their concerns and fear that they lacked the knowledge to make such an important decision, which resulted in the clinician helping them to eliminate these concerns or increase their knowledge by arranging an appointment with another professional, e.g. a radiation or medical oncologist. Some patients reported that they had a firm intuitive preference from the beginning, which did not change throughout the process.

*‘That I would have to face the consequences of the radiation therapy really bothered me, and I really didn’t feel that I had to keep my breast at all costs. To make sure that I was well informed anyway, the surgeon made an appointment with the radiation therapist, so I could consult with her as well to help me make my decision.’ Patient 7*

### Decision talk

Although most patients did not report any explicit decision talk and some patients reported a more paternalistic style of decision talk, patients felt that, eventually, they made their own decision. The majority of the patients felt no pressure and were given time to think and talk to relatives about the options. A few days later they were given a follow-up appointment, often with a breast cancer nurse to reconsider or confirm their first intuition on treatment. Four patients stated they did not make the decision, either because they felt they had no choice or felt they had no preference, nor the knowledge to make such an important decision.

*‘Well, his first choice was breast-conserving therapy, and … which woman wouldn’t want that, I guess, but then he did add that I also had the choice of breast amputation. He said I had a choice, but that in principle it wasn’t necessary.’ Patient 13*

*‘I simply don’t have the knowledge. I just have to assume – and want to assume – that they have my best interests at heart.’ Patient* 2

#### Perceived support of the PtDA.

At the time of the interview, 13 (65%) of the patients had been using the PtDA. The non-users were all educated at an intermediate or low level. Reasons for not using the PtDA were; no time (*n* = 2), received enough information (*n* = 2), no computer skills (*n* = 2), SDM too difficult. The patients who used the PtDA thought the information was clear and useful. Most of them remembered what they read and how they scrolled through the PtDA. Some patients had scrolled through the website reading bits and pieces they found interesting, while others had only read the screens that applied to them. Most of the patients indicated that it had been useful to read all the information again. They recognised most of the information they had already received verbally, but reading this again encouraged them to rely on their first intuitive preference or to feel confirmed in their deliberation about the treatment decision. One patient used the PtDA and decided to request a new appointment with her surgeon, after which she changed her treatment decision.

Half of the patients who used the PtDA stated they had read and completed the value elicitation part of the PtDA. A majority of the patients who had completed the value elicitation exercise had printed the summary of the answers they had given with the aim of presenting it during the follow-up consultation with the breast cancer nurse. Only one breast cancer nurse asked to look at and talk about it. Patients who had printed the summary and taken it to the next consultation were disappointed because the clinician did not inquire after their preferences. Two patients found completing the value elicitation statements emotionally distressing.

None of the patients viewed the PtDA as the primary resource for their treatment decision, although it had helped them become better informed. Patients regarded their surgeon and family as their most important decision-making resources. Most patients did not particularly use the PtDA to prepare the decision talk with their clinician.

*‘Well, I did make a printout and took it along, but we didn’t discuss it specifically.’ Patient 18*

*‘So that was clear. Fine. Although I think that at that point I had more or less decided, I did appreciate the opportunity to read it all again.’ Patient 16*

*‘I did complete the decision aid and it had me thinking: well, they can depict it like that, and I’m seeing it all on screen, but I prefer to hear it from someone. Matters were pretty clear and when I completed the decision aid I did think: well, I’ll go for breast-conserving therapy, that’s what I’ll do. But I still wanted to discuss it with the doctor … and that’s what really decided matters in the end.’ Patient 9*

## Discussion

### Summary of main findings

With regard to the SDM process, patients felt that clinicians made an effort to inform and involve them, which resulted in patients being satisfied with the process and having experienced SDM. Choice talk and option talk was occasionally somewhat suggestive and not always completely refrained from the clinician’s implicit opinion, but patients felt well-informed and felt they had explicitly decided on the treatment option. Elicitation of preferences was very limited, both during the first and the follow-up consultation, after patients had been exposed to the PtDA. Apparently, a clear structure in the second half of the SDM process was lacking. Most patients did not remember any explicit value clarification or decision talk.

In general, breast cancer nurses delivered and explained the PtDA to patients. A majority of the patients, mostly patients with a high level of education used, appreciated the PtDA and thought it was useful especially to recall all information given, but patients used the PtDA in various ways. Some patients read all the available information, completed the value elicitation exercise and read and printed the summary, while other patients just read one chapter and only briefly looked at the value elicitation questions. Patients that took the summery to the successive consultation were sometimes disappointment or surprised that the breast cancer nurse never asked to discuss it.

### Strengths and limitations

The qualitative design of this study enabled us to learn about the experiences of patients with regard to SDM and the usefulness of the PtDA. The sample size of this study (*n* = 20) was sufficient to reach data saturation. No new themes emerged from the data after 17 interviews. Although we urged the breast cancer nurses to recruit all the patients who were offered a PtDA, selection bias might have occurred with regard to patients who were most willing to use the PtDA. Originally, we planned a pre- and post-implementation study measuring objective and subjective results on outcome and process. Due to a difficult inclusion of patients, the quantitative data were too scattered to use as evidence [20]. However, the data were meaningful to use as a source to enrich the qualitative work, helping us to get to the core questions and dig deeper in the memory of patients. The number of patients included in the interviews per participating hospital was not evenly distributed. One hospital supplied only one patient for this study, while another hospital supplied six patients.

This pilot study was part of a larger study on the implementation of SDM with the purpose of studying both patient and professional point of view on intention-behaviour gaps. Barriers to implement SDM from clinicians were not taking into account in this part of the study.

### Comparison of the results with the current literature

In general, patients were positive about the perceived level of SDM and felt well-informed, which is reflected in the results of SDM-Q9 and CollaboRATE. True patient-centeredness was experienced especially when health care professionals showed themselves to be knowledgeable about personal details. This finding is consistent with conclusions from other studies, in which it became clear that the degree of trust in their clinician contributes to patients’ feeling of being well-informed [32, 33]. The finding of patients not experiencing preference elicitation or value clarification during the medical encounters was in line with objective data we gathered.

Although the efforts of breast cancer nurses, who were viewed as easily approachable case managers, were highly appreciated by patients, their role varied considerably between hospitals and patients. In some hospitals they played a major part in the consultation; they were deployed to educate and counsel patients, in other hospitals their role was limited to coordinating the care process. This was also consistent with findings in other studies [34]. Nurses often also performed the last consultation before the patient had surgery. We recommend that breast cancer teams review their roles in the pathway, define them, and give nurses a clear responsibility in SDM, especially with regard to eliciting preferences.

Patients were overwhelmed by the amount of both verbal and written information they received during the encounters, especially the first one. They not only received information about their diagnosis and treatment but also on hospital regulations, follow-up appointments, specific support and research projects most of the breast cancer units are involved in. We recommend that all the information given to patients is reviewed. Some of the written materials may not be applicable right after diagnosis and could be handed over during the second consultation, after the patient has had time to use the PtDA.

Concerning the PtDA, there was some variation in use and satisfaction. The percentage of patients using the PtDA corresponded to results from other studies [35]. Patients that used the PtDA thought it was useful, especially in recalling the information they received from the clinician. Reading the information again strengthened them in underpinning their deliberation about the treatment.

## Conclusion

In this study, we explored patients’ experiences on SDM and a recently developed PtDA. It seems obvious that the SDM process is taking small steps forward. We found a suitable level of perceived SDM, in the first two steps (choice and option talk) of the SDM process. Patients felt well-informed, reported being involved in the decision making process and were satisfied with the process and the decision made. Nevertheless, the perceived level of SDM was largely based on whether the first two steps of SDM, choice talk and option talk, were performed satisfactorily. Decision talk, in which preferences should be explored, remained limited. This implies that in the implementation of SDM, more focus is needed on this part of the process. We may need to clarify to clinicians that SDM means not only transmitting information, but also engaging in a dialogue in which patients’ preferences are discussed. Together with clinicians, we should define who performs this dialogue and when it should take place in the care pathway. This important part of the process could be assigned to well-educated breast cancer nurses with sufficient knowledge of SDM. Finally, a clear explanation on how to use the PtDA, including the value clarification, and motivating patients to use the PtDA as intended is necessary to persuade more patients to use the PtDA.

## Data Availability

All the data sets collected and analysed during the current study are available from the corresponding author on reasonable request.

## Appendix

### Interview guide

Thank you for taking the time to speak to me today. You have agreed to be interviewed for this study about shared decision-making in clinical practice. With your permission, the interview is being recorded because we need to transcribe it. As agreed, everything that is discussed today will be processed anonymously and your name will not be mentioned in the report.

I would like to ask you some questions about your experiences with shared decision-making and the patient decision aid. Do not hesitate to tell me if you do not understand something. There are no good or bad opinions, you can say whatever you like. The results of this interview will be used to learn more about the implementation of shared decision-making and the usefulness of a patient decision aid.**In general**

1. Could you describe the consultation during which you received the diagnosis and the treatment options/treatment plan?
2. How did you experience the conversation with your surgeon and/or your nurse/nurse practitioner in general?
3. Please describe how you and your surgeon determined a treatment plan. How did you decide on a mastectomy/breast-conserving therapy?
4. How did you experience the decision–making process?

- What information did you receive? What did you think of the information you received? At that time, did you understand the information?
- Were you able to contribute to the conversation? If so, what was your contribution?
- Were you able to express your preferences? If so, how did you express your preferences and how did the surgeon respond?
- How was the decision made? (you, your surgeon, together)

5. What could have been done better?

**Patient decision aid (PtDA)**

6. Did you use the PtDA? If no, why not?

If yes:

7. How did you appreciate the PtDA in general?
8. How did you appreciate the information you read in the PtDA?

- Did you find the information you read useful?
- Did you find the information in the PtDA neutral?
- Did you find the information complete?
- What was your opinion with respect to the amount of information?
- Did you read all the information? If not, which parts did you read?

9. Did you read the value clarification statements? If yes, did you use them according to the explanation? Did you print the summary? Did you take the summary to your next consultation?
10. Did you find the PtDA feasible?

**Patient decision aid – implementation**

11. What is your opinion with regard to the timing of the delivery of the PtDA? How and by whom was the PtDA delivered? What did you appreciate in this process?
12. Would you rather have another clinician delivering the PtDA or done at another time?
13. Did the PtDA influence the knowledge you had about breast cancer and the treatment?
14. Did the PtDA influence your treatment decision?
15. How do you feel about the treatment you chose?

Those were my questions. If there is anything you would like to add to this interview, now is the time to do so. If not, I would like to thank you for your time and the openness that you have shown me. We will send you a report of this interview. You can read it and see whether you agree or not. If you do not agree or have remarks, you can email me. If you do not respond within 3 weeks, I assume you agree with how our conversation has been represented.

## Abbreviations

SDM: Shared Decision Making
PtDA: Patient Decision Aid
MUMC+: Maastricht University Medical Centre
SDM-Q9: Shared Decision Making Questionnaire
DCS: Decisional Conflict Scale
